# Adverse maternal outcomes of adolescent pregnancy in Northwest Ethiopia: A prospective cohort study

**DOI:** 10.1371/journal.pone.0257485

**Published:** 2021-09-22

**Authors:** Getachew Mullu Kassa, Ayodele O. Arowojolu, Akin Tunde A. Odukogbe, Alemayehu Worku Yalew

**Affiliations:** 1 Department of Obstetrics and Gynaecology, Pan African University Life and Earth Sciences Institutes, College of Medicine, University of Ibadan, Ibadan, Nigeria; 2 College of Health Sciences, Debre Markos University, Debre Markos, Ethiopia; 3 Department of Obstetrics and Gynaecology, College of Medicine, University College Hospital, University of Ibadan, Ibadan, Nigeria; 4 School of Public Health, College of Health Sciences, Addis Ababa University, Addis Ababa, Ethiopia; University of Botswana, BOTSWANA

## Abstract

**Background:**

Adolescent pregnancy is considered a major contributor to maternal and child morbidity and mortality, the greatest concern of developing countries and an important public health issue globally. Adolescents are responsible for eleven percent of births worldwide and they face several pregnancy and childbirth related complications. However, in low-income countries like Ethiopia, there are limited researches conducted to investigate outcomes of adolescent pregnancy. Therefore, this study was conducted to assess the adverse maternal outcomes of adolescent pregnancy in Northwest Ethiopia.

**Methods:**

A prospective cohort study was conducted in 12 health facilities from seven districts in East Gojjam zone, Northwest Ethiopia. A total of 418 adolescents (15–19 years old) and 836 adult women (20–34 years old) who attended randomly selected health facilities in East Gojjam zone were included. Data were collected starting from admission to the maternity ward for labor and delivery, and postnatal depression was measured at six weeks’ postpartum period using the Edinburgh Postnatal Depression Scale. Generalized estimating equations (GEE) was used to account for the within subject correlation and assess the effect of different known factors that could influence the outcome of this study.

**Results:**

A lower percentage of adolescent (58.4%) than adult (71.2%) women had their first antenatal care booking before 16 weeks of gestation. After adjusting for different confounding factors, the adverse outcome that was significantly associated with adolescent pregnancy was postpartum depression (AOR: 2.29; 95% CI, 1.42, 3.7, *p-value = 0*.*001)*. Assisted vaginal delivery (AOR: 0.44; 95% CI, 0.23, 0.86, *p-value 0*.*016)* and cesarean section (AOR: 0.43; 95% CI, 0.19, 0.97, *p-value = 0*.*042)* were significantly lower among adolescent women.

**Conclusions:**

Adolescent pregnancy is associated with higher odds of postpartum depression, and lower odds to undergo cesarean section and assisted vaginal delivery than adult women. Perinatal care services should be more adolescent-friendly to ensure early diagnosis and treatment of postpartum depression. School and community-based awareness programs regarding use of contraception to prevent unwanted adolescent pregnancy, early antenatal care booking and adverse pregnancy outcomes of adolescent pregnancy and provision of psychosocial support are recommended.

## Introduction

Adolescents-aged 10 to 19- are considered as the most important population group for reduction of reproductive health problems in developing countries [1]. Most adolescents start sexual intercourse early [2], which leads to unwanted pregnancy and other sexual and reproductive health (SRH) problems [3]. Adolescents account for 11% of births worldwide [4], and they face several SRH problems related to pregnancy and childbirth [2]. Adolescent pregnancy is a major contributor to maternal and child morbidity and mortality and the cycle of poverty, which is of great concern to developing countries [5,6].

One in five adolescent girls start childbearing in Africa, the highest magnitude being in the East African region (21.5%) [1]. The main factors responsible for the high rate of adolescent pregnancy in sub-Saharan Africa (SSA) include individual factors, sociocultural, economic and health service-related factors [7]. Particularly, rural residence, early marriage, not attending school, no maternal or paternal education, and absence of parent-to-adolescent communication on SRH issues were the main factors associated with adolescent pregnancy in Africa [1]. Due to the maternal and newborn complications associated with adolescent pregnancy, prevention of pregnancy among young girls in SSA has become one of the major focuses of international organizations [8].

In Ethiopia, 13% of adolescent girls have already started childbearing [9]. The percentage of adolescents who started childbearing is higher among those living in rural areas (14.8%) than those in urban areas (4.9%). The prevalence of adolescents bearing children was also highest among those in the lowest wealth quantiles (24%) than those in the highest wealth quantile (5.8%) [9]. Moreover, the main factors responsible for the high rate of adolescent pregnancy in developing countries are related to cultural beliefs, child marriage and poor access to contraceptive information and services [10].

Complications related to pregnancy and childbirth are considered the leading causes of death among adolescents in developing countries [11]. More than half of maternal deaths globally are because of hemorrhage, pregnancy related hypertension and sepsis [12]. In Ethiopia, the main causes of maternal mortality are obstructed labor, hypertensive disorders and hemorrhage [13]. These types of complications are more common among adolescent than adult women. For example, hypertensive disorders of pregnancy are higher among adolescent than in older women [14]. Studies conducted in Nigeria, Cameroon, and Brazil also showed higher risk of hypertensive disorders among adolescents than adult women [15–17]. Therefore, a focus on adolescent pregnancy will help to reduce the high rate of maternal and child morbidity and mortality [1]. Moreover, prevention of adolescent pregnancy is considered as one of the main intervention strategies to improve women’s education and to improve social and economic development of a country [18].

Previous studies showed inconsistent findings in terms of the adverse maternal outcomes of adolescent pregnancy. For example, a large, intercontinental population-based cohort study conducted in low- and middle-income countries showed non-significant differences in the risk of adverse maternal outcomes among adolescents compared to youths aged 20–24 years [4]. On the other hand, a study conducted in Zambia showed a higher risk of eclampsia, anemia, vaginal bleeding and prolonged labor among adolescent than older women [19]. Another study [20] showed a decreased risk of maternal complications like preeclampsia, post-partum hemorrhage (PPH), and caesarean section among younger than adult women.

There are limited studies in Ethiopia assessing adverse maternal outcomes of adolescent pregnancy, including postpartum depression (PPD). A systematic review study that was conducted on this topic also recommended the need of studies addressing this issue [21]. In addition, there are limited studies conducted to assess the adverse maternal outcomes of adolescent pregnancy including postpartum depression in Ethiopia.

Therefore, the current study was conducted to assess the adverse maternal outcomes of adolescent pregnancy in Northwest Ethiopia. Outcomes that were not covered in previous studies such as PPD were also included in this study. The use of a prospective follow-up design to collect data on postpartum complications in this study will also provide a special contribution to the area of adolescent reproductive health. Moreover, identification of the adverse maternal outcomes of adolescent pregnancy in Ethiopia will assist policy makers and other stakeholders in designing proper strategies to prevent these complications.

## Materials and methods

### Study area, design and period

This study was conducted in selected health facilities of East Gojjam zone, Amhara region, Northwest Ethiopia. The main administrative city of East Gojjam zone is Debre Markos, which is located 300 km Northwest of Addis Ababa, capital city of Ethiopia. According to the 2017 population projection estimate, the zone had a total population of 2,153,937. Of this, 1,066,716 were men and 1,087,221 were women. The majority (90%) of the East Gojjam zone inhabitants live in rural areas, while 213,568 are urban inhabitants [22]. According to the 2016/17 annual report of East Gojjam zone health office, there were nineteen districts in the zone, and 102 health centers, eight primary hospitals and one referral hospital in the zone. The total number of pregnant mothers attending at least first ANC follow up in the zone are 87,044. In addition, the annual report (July 2016 to June 2017) of the zone showed that the number of pregnant mothers who delivered at health institutions was 78,229, resulting in 90% institutional delivery rate [23]. A prospective cohort study was used to assess the adverse maternal outcomes of adolescent pregnancy. This study was conducted from January 3/2018 to December 25/2018.

### Population and eligibility criteria

The study population included all pregnant adolescent (aged 15–19) and pregnant adult women (aged 20–34) who visited randomly selected health facilities in the study area. Women with multiple pregnancies (more than one) were excluded from this study as it could affect the pregnancy outcomes [24,25].

### Sample size and sampling procedure

Double population proportion formula for cohort study was used to calculate the sample size [26]. Assumptions included 80% power, 95% confidence interval (CI), 2 to 1 ratio of adult to adolescent women, a design effect of 2, and 10% non-response rate/ lost from follow up [27]. Sample size was calculated using different adverse maternal and neonatal outcomes from various studies, and the highest sample size was obtained using low Apgar score at first and five minutes after birth outcome from a study conducted in Addis Ababa, Ethiopia [28]. Accordingly, a total of 1,254 participants (418 adolescents and 836 adult women) were included in this study.

Multistage sampling technique was utilized to select representative sample of districts and health facilities in the study area. Seven out of nineteen (37%) districts were randomly selected in the zone using simple random method. Then random selection of twelve health facilities was done from the selected district in the study area. Proportional sample size allocation was made to each district and to selected health facilities based on their previous annual client flow. Adolescent women were included in the study consecutively as they came to the health facility for delivery, while the next two adult women after each selected adolescent were included as controls

### Data collection methods

Data collection tool was prepared after review of guidelines, survey instruments and related literatures on adolescent pregnancy and its adverse outcomes [29–42]. Pretest was conducted and all necessary amendments were made on the research questionnaire before the actual data collection period. Training was also provided to data collectors and supervisors involved in the data collection and supervision process. The training focused on objectives of the study, data collection tool, ethical issues, and overall data collection process.

Study participants were recruited when they come to the selected health facilities. Data were collected from the mother and the neonate of adolescent and adult women in labor ward, postnatal ward and at home in the postpartum period. The hospital-based data collection was made by health care workers (midwives and nurses) in the selected health facilities. The hospital-based data collection involved both primary and secondary data sources. Both interviews administered data collection and data collection from the medical cards and clinical charts were used. Community Health Extension workers (CHEWs) assisted the community-based data collection at six weeks after delivery. Completeness and accuracy of the collected data were checked on a daily basis by research supervisors and the Principal Investigator.

### Variables of the study and definition of outcomes

Several sociodemographic variables, obstetric factors, substance use and medical related factors were included as independent variables. The socio-demographic variables included age, residence, religion, ethnicity, educational status, occupation, living arrangement, educational and occupational status of the mother and the father, marital status, and wealth index. The obstetric factors include antenatal care (ANC) service utilization, number of visits, time of ANC initiation, immunization, iron/folic acid supplementation, gravidity and parity, pregnancy planning, violence against women during pregnancy, and postnatal care (PNC). Substance use like Alcohol, Smoking, *Khat*, and use of other drugs were also included in the study. In addition, medical factors like family history of Diabetes Mellitus (DM) and hypertension, previous history of medical and surgical conditions, a distance of health facility from home, and nutritional status were also included in the study.

The outcome of this study was adverse maternal outcomes of adolescent pregnancy. Adverse maternal outcomes included in this study include the following problems: preeclampsia, premature rupture of membranes (PROM), postpartum hemorrhage (PPH), and PPD. Preeclampsia is defined as diastolic blood pressure greater than or equal to 90 mmHg and systolic blood pressure of 140 mmHg or more recorded on two occasions, 4 hours apart (except when initial readings are above 150 mmHg systolic and 100mmHg diastolic) and +1 dipstick proteinuria after 20 weeks of gestation for a woman who is previously normotensive [43,44].

Postpartum depression was measured using the Edinburgh Postnatal Depression Scale (EPDS) at six weeks postdelivery [45,46]. The tool contains ten items to assess depression. The score ranges 0 to 3 for each item, which indicates from no to higher severity of depression. Study participants whose EPDS scores were more than or equal to 13 ponts were categorized as having depression, and those below 13 were grouped as no depression [45,46]. Moreover, EPDS tool was previously validated [47] and used in studies conducted in Ethiopia [42,48,49].

In addition, the use of assisted vaginal delivery (vacuum extraction or forceps delivery), and cesarean section were assessed and compared between the two population groups. Assisted vaginal delivery is defined as “the use of instruments like forceps or vacuum devices to assist the mother in effecting vaginal delivery of a fetus” [50]. Gender based violence (GBV) was also mesured in this study. The questions were adapted from the Ethiopian Demographic and Health Survey (EDHS) 2016 to measure GBV in the current study [51]. It is described as physical, sexual and psychological abuses of the sufferer. It was measured among adolescent and adult women. Study participants were asked if they had experienced any form of gender based violence (physical, sexual, or psychological violence) during the current pregnancy. The responses were summed and a summary index was created for any form of gender based violence during current pregnancy.

### Data management and analysis

Collected data were entered using EpiData (version 3.1; Denmark) software and was exported to STATA software (version 14; StataCorp, College Station, TX) for further analysis. Descriptive and inferential statistics were conducted to describe the population characteristics in relation to the outcome variables. Categorical outcomes were compared using chi-square test, and independent t-test was used for comparison of the mean differences of continuous variables between adolescent and adult women.

In addition, Generalized Estimating Equations (GEE) were used to determine the relationship of adolescent pregnancy with adverse maternal outcomes after adjusting for known factors. The model adjusted for different factors like: residence, school attendance, wealth status, history of previous pregnancy, antenatal care (ANC) follow up, iron-folic acid supplementation during current pregnancy, hypertension, anemia, and GBV during current pregnancy (physical, sexual or psychological violence). For instance, for the PPD outcome, the final model adjusted for the following several variables like age of the mother, residence, marital status, school attendance, wealth status, anemia, iron folic acid supplementation, gender-based violence during pregnancy, postnatal care attendance, pregnancy status (wanted vs not wanted), preeclampsia, 24- hour dietary diversity, and social support (low, medium and high).

Moreover, GEE is essential to control within subject correlation and intra-class correlation that may have been introduced in this study [52]. The findings were presented using Adjusted Odds Ratio (AOR) and its 95% confidence intervals (CI).

### Ethical consideration

The Institute for Advanced Medical Research and Training (IAMRAT), College of Medicine, University of Ibadan, Ibadan, Nigeria approved this study. The I/UCH EC Registration Number is NHREC/05/01/2008a and UI/UCH Ethics Committee assigned number is UI/EC/17/0440. The study was also approved by Debre Markos University research ethics committee. Support letter was written from Amhara Public Health Institute to east Gojjam Health office, Debre Markos, Ethiopia. Written informed consent or assent was obtained from each study participant, and parents/guardian of those less than 18 years before data collection. The consent was obtained after explaining the purpose of the research, the data collection procedures, the benefits and risks of participating in the study and the voluntariness of participation. Collected data was treated with utmost confidentiality.

## Results

### Sociodemographic characteristics

**Fig 1:**
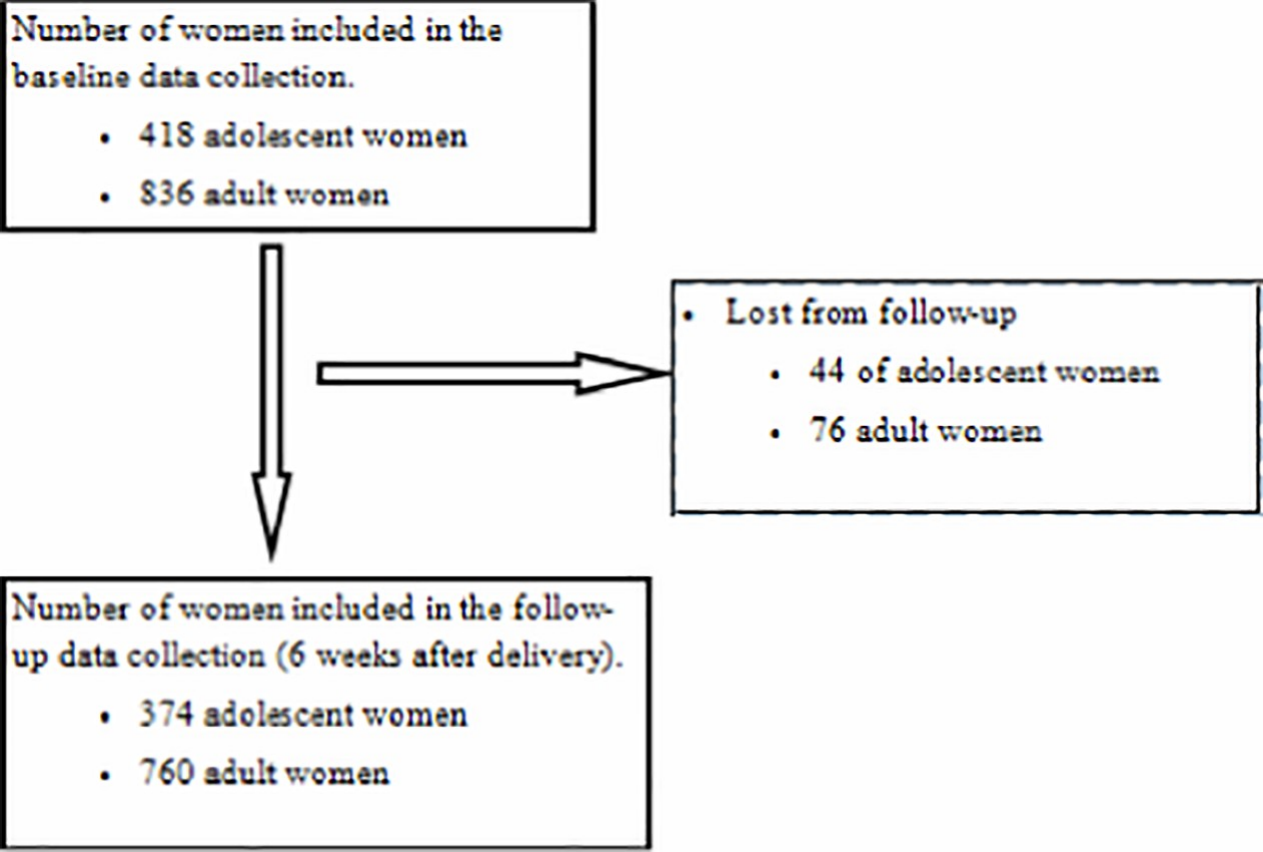
Study flow diagram of enrollment into the study and follow-up.

This study included 1,134 study participants, accounting for 90.4% of the total sample size. A total of 374 out of 418 (response rate = 89.5%) adolescents aged 15–19 and 760 out of 836 (response rate = 90.9%) adult women aged 20–34 completed the study (Fig 1). More than half (57.2%) of adolescent population were from rural area, while 44.7% of adult women were from rural area. More than two-thirds (72.2%) of adolescents ever attended school. From these, 70% of adolescents had only primary education and 23% attended secondary school. Amongst adult women, 39.7% attended only primary school and 32.7% had secondary education. Majority, 95% and 98.3%, of adolescent and adult women, respectively, were currently married. One-fifth (19.6%) of adolescents and 22.7% of adult women were in the lowest wealth quantile.

### Reproductive and obstetric characteristics

A large percentage (60.4%) of adolescent women were married before their eighteenth birthday compared to 33.5% of adult women. More of adults (95.5%) than of adolescent women (88%) attended antenatal care for the current pregnancy. In addition, a lower percentage (58.4%) of adolescent than adult women (71.2%) booked for antenatal care before 16 weeks of gestation. The total number of ANC visits among the two groups was significantly different. The mean number of ANC visits was 3.26 ± 1.24 for adolescent women and 3.54 ± 1.5 for adult women, *p-value = 0*.*003*) (Table 1).

**Table 1:** Selected reproductive and obstetric characteristics of women who gave birth at public health facilities in East Gojjam zone, Northwest Ethiopia, 2018.

Variables		Adolescents (15–19 years old) n (%)	Adults (20–34 years old) n (%)	*p-value*
Age at first marriage (in years)	Less than 18	218 (60.4)	240 (33.5)	*<0*.*0001*
	18 and above	143 (39.6)	476 (66.5)	
Time of first ANC booking	Less than 16 weeks	180 (58.4)	468 (71.2)	*<0*.*0001*
	After 16 weeks	128 (41.6)	189 (28.8)	
Number of ANC visits during the last pregnancy	(mean+SD)	3.26±1.24	3.54±1.5	*0*.*003*
Partner came to health facility for ANC purpose during the current pregnancy	No	178 (52.8)	314 (42.5)	*0*.*002*
	Yes	159 (47.2)	425 (57.5)	
Physical violence during current pregnancy	No	347 (92.8)	721 (94.9)	*0*.*158*
	Yes	27 (7.2)	39 (5.1)	
Sexual violence during current pregnancy	No	347 (93)	732 (96.3)	*0*.*015*
	Yes	26 (7.0)	28 (3.7)	
Psychological violence during current pregnancy	No	315 (84.5)	718 (94.5)	*<0*.*0001*
	Yes	58 (15.5)	42 (5.5)	
Experienced Gender Based Violence (GBV) during current pregnancy	No	306 (82)	686 (90.3)	*<0*.*0001*
	Yes	67 (18)	74 (9.7)	

Concerning experiences of gender-based violence in current pregnancy by the two groups, more adolescents reported this episode than adults, irrespective of the type. The percentage of physical violence (7.2 vs. 5.1, *p-value = 0*.*158*) was higher among adolescent than adult women. Similarly, the percentage of sexual (7.0 vs 3.7, *p-value = 0*.*015*), and psychological violence (15.5 vs 5.5, *p-value <0*.*0001*) were significantly higher among adolescent than adult women. Overall, the percentage of at least one form of GBV was higher among adolescent (18%) than adult women (9.7%), and this was statistically significant, *p-value <0*.*0001* (**Table 1**).

### Adverse maternal outcomes

**Fig 2:**
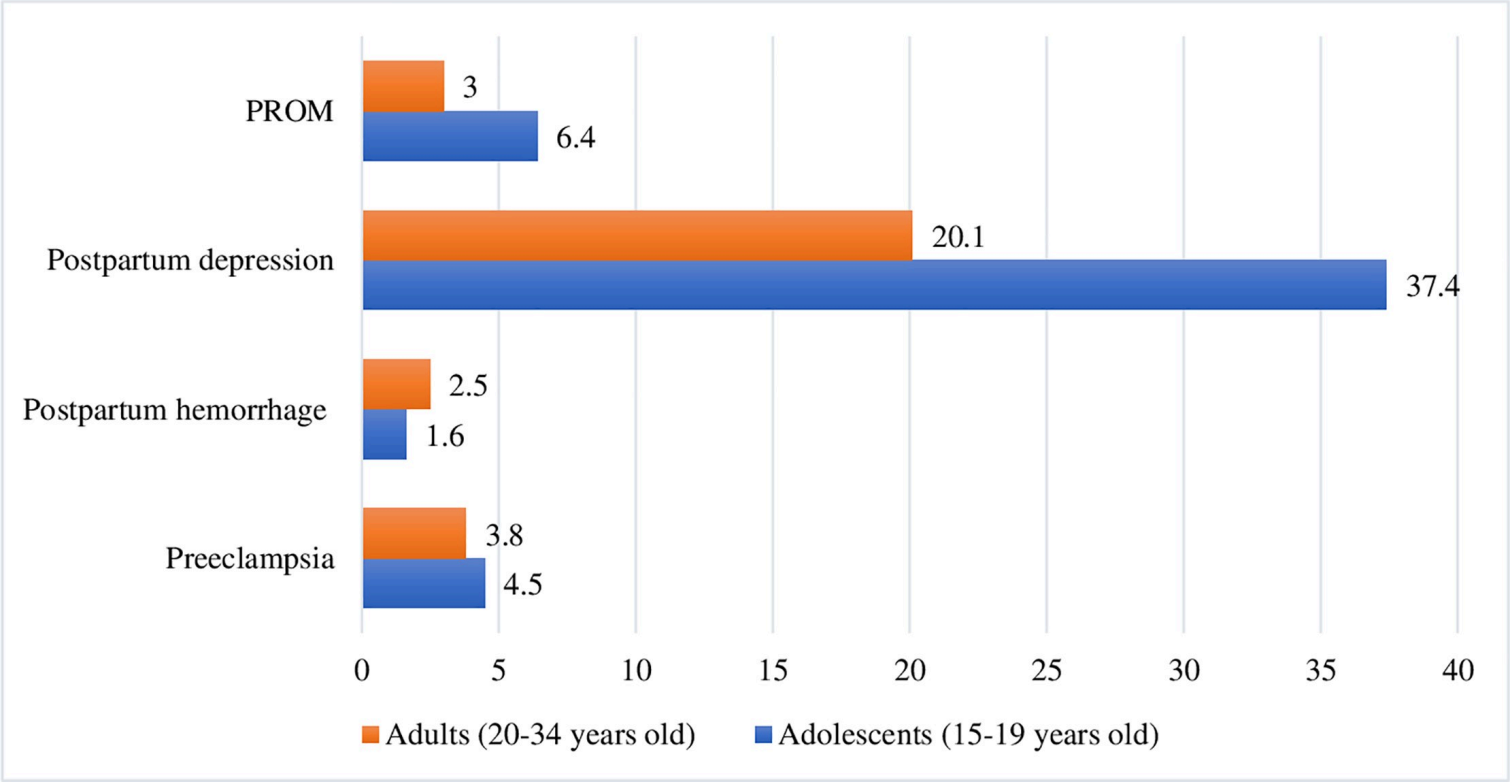
Percentage distribution of adverse maternal outcomes among women who gave birth at public health facilities in East Gojjam zone, Northwest Ethiopia, 2018.

There were variations observed in the percentages of adverse maternal outcomes between adolescent and adult women. Seventeen (4.5%) of adolescent women had preeclampsia compared to 29 (3.8%) of adult women, although the difference was not statistically significant (*p-value = 0*.*558*). The percentage of postpartum hemorrhage among adolescent women was lower than in adult women (1.6% vs. 2.5%, *p-value = 0*.*334*), but this was not significant. However, a significantly higher percentage of adolescent women had PPD (37.4%) compared to adult women (20.1%), *p-value <0*.*0001*. In addition, the proportion with premature rupture of membranes was also significantly higher among adolescent than adult women (6.4% vs. 3%, *p-value* = *0*.*007*) (Fig 2).

A large proportion (30.5%) of adolescent women had episiotomy compared with adult women (10.9%). On the other hand, the percentage of adult women who had cesarean section (CS) was higher (11.2%) than adolescent women (6.7%). The most common indication for CS among both adolescent (44%) and adult women (33.3%) was Non-Reassuring Fetal Heart Beat (NRFHB). The percentages having cephalopelvic disproportion (CPD) as indication for CS among adolescent (16%) was higher than among adult women (3.6%) (Table 2).

**Table 2:** Adverse maternal outcomes among women who gave birth at public health facilities in East Gojjam zone, Northwest Ethiopia, 2018.

Outcomes	Percentage (95% CI)	
	Adolescents (15–19)	Adults (20–34)
PROM	6.4 (4.2, 8.9)	3 (1.9, 4.3)
PPD	37.4 (32.6, 41.9)	20.1 (17.1, 22.9)
PPH	1.6 (0.5, 3.1)	2.5 (1.5, 3.7)
Preeclampsia	4.5 (2.5, 6.7)	3.8 (2.6, 5.3)
Cesarean section	6.7 (4.4, 9.4)	11.2 (8.8, 13.4)
Indications for Cesarean section		
CPD	16 (4, 32)	3.6 (0, 8.4)
Failure to progress in labor	20 (4, 36)	12 (6, 19.3)
Preeclampsia	8 (0, 20)	6 (1.2, 12)
Fetal malpresentation	12 (0, 24)	19.3 (10.8, 27.7)
Fetal malposition	4 (0, 12)	13.3 (6, 21.7)
NRFHB	44 (24, 64)	33.7 (24.1, 43.4)
Others ^a^	16 (4, 32)	25.3 (15.7, 34.9)
Episiotomy	30.5 (25.8, 35.1)	10.9 (8.8, 13.3)
Forceps delivery	1.1 (0.3, 2.5)	2.6 (1.6, 3.8)
Vacuum delivery	7.0 (4.4, 9.9)	4.6 (3.2, 6.3)
Assisted vaginal delivery	8.0 (5.5, 11.0)	7.2 (5.5, 9.2)

^a^ previous CS scar, post-term pregnancy, oligohydramnios, obstructed labor, vaginal, vulval and perineal tear, failed induction of labor, prolonged labor and abruption placenta.

After adjusting for different confounding factors, the adverse outcomes that was significantly associated with adolescent pregnancy was PPD. The odds of developing PPD among adolescent women were two times higher than among adult women (AOR: 2.29; 95% CI, 1.42, 3.7, *p-value = 0*.*001)*. In addition, assisted vaginal delivery (forceps delivery or vacuum extraction) was significantly different amongst the two groups. Adolescent women had 57% less odds to undergo cesarean section than adult women (AOR: 0.43; 95% CI, 0.19, 0.97, *p-value = 0*.*042)*, 81% less odds for forceps delivery (AOR: 0.19; 95% CI, 0.12, 0.34, *p-value <0*.*0001)*, and 56% less odds to undergo assisted vaginal delivery (AOR: 0.44; 95% CI, 0.23, 0.86, *p-value 0*.*016)*. However, adverse outcomes like preeclampsia, postpartum hemorrhage, and PROM were not statistically significant to occur with younger maternal age (Table 3).

**Table 3:** Results of GEE analysis to identify the adverse maternal outcomes of adolescent pregnancy in Northwest Ethiopia, 2018.

Adverse outcomes	AOR (95% CI)	*p-value*
Preeclampsia		
Adolescents (15–19 years old)	1.25 (0.76, 2.06)	*0*.*383*
Adults (20–34 years old)	Reference	
Postpartum hemorrhage		
Adolescents (15–19 years old)	0.69 (0.32, 1.48)	*0*.*34*
Adults (20–34 years old)	Reference	
Postpartum depression		
Adolescents (15–19 years old)	2.29 (1.42, 3.7)	*0*.*001*^*b*^
Adults (20–34 years old)	Reference	
Premature rupture of membrane (PROM)		
Adolescents (15–19 years old)	1.69 (0.88. 3.24)	*0*.*11*
Adults (20–34 years old)	Reference	
Cesarean section		
Adolescents (15–19 years old)	0.43 (0.19, 0.97)	*0*.*042*^*a*^
Adults (20–34 years old)	Reference	
Assisted vaginal delivery		
Adolescents (15–19 years old)	0.44 (0.23, 0.86)	*0*.*016*^*a*^
Adults (20–34 years old)	Reference	

^a^ significant at p <0.05;

^b^ significant at p <0.0001.

AOR (95% CI): Adjusted Odds Ratio at 95% confidence level.

The model adjusted for different factors like; residence, school attendance, wealth status, history of previous pregnancy, antenatal care (ANC) follow up, iron-folic acid supplementation during current pregnancy, anemia, and GBV during current pregnancy (physical, sexual or psychological violence).

## Discussion

This study was conducted to assess the adverse maternal outcomes of adolescent pregnancy in Northwest Ethiopia. This study found a higher odd of PPD among adolescent than adult women. The odds of cesarean section and assisted vaginal delivery were found to be significantly lower among adolescent than adult women.

Adolescent women were more than twice at higher odds of developing PPD than adult women in the current study. The postpartum period is a risky time for the development of severe mental mood disorders, and it affects 10 to 15% of childbearing women [53]. Particularly, depression is one of the leading causes of disability among adolescents and is considered an important public health issue. Postpartum depression occurs almost three times more commonly in developing countries than developed countries [54]. According to the 2016 World Health Organization (WHO) report, PPD is the third leading cause of illness in the younger population [55]. Younger maternal age was also found to be significantly associated with PPD at six weeks in a study conducted Uganda [54].

Postpartum depression is associated with higher risk of morbidity and suicide risk among adolescents [56], and increases the occurrence of maternal and child complications during the postnatal period [40]. Previous study has also shown a higher percentage of PPD among adolescents than adult women [57]. A higher odds of PPD among adolescent women could be because many adolescents are disadvantaged before becoming pregnant [58], which results in physical, psychological, and economic challenges during pregnancy and following childbirth [59]. In addition, most adolescent mothers do not graduate from high school compared to non-pregnant adolescents, only 40% from pregnant and 75% non-pregnant adolescents (who delay first birth up to 20–21 years old) complete high school [60].

Postpartum depression impedes maternal ability to care for the child in the first year of infant’s life. It affects the physical health of the newborn child, predisposing to infectious and nutritional diseases and impaired physical growth [61]. For example, a study conducted in Pakistan showed that infants of pre–and postnatally depressed mothers are at higher risk of developing underweight, stunting, diarrhea, and emotional and behavioral problems [62]. In addition, poor maternal-infant attachment/ bonding is also another problem associated with PPD [63].

Pregnancy during adolescence also causes a broad range of social and psychological imbalances [64]. Psychosocial problems other than obstetric and medical risks related to adolescent pregnancy have higher impact on the mother, families and the community at large [65]. They are associated with poor educational achievement and school drop out or vocational training, which result in limited future job opportunities [66,67]. Hence, they are related to limited life options, economic disadvantage, social isolation, and stigma [68–71].

Therefore, prevention, early identification and treatment of depression and other psychosocial issues among adolescent women during antenatal follow up and the postnatal period is essential to reduce the adverse effects on maternal and infant health. Pregnant adolescents should be considered as a target group for psychological counselling and support related to mental health during pregnancy and in the postnatal period. The presence of family conflict, lack of or inadequate social support and low level of self-esteem were associated with PPD among adolescent women in a previous study [72]. Intervention programs need to focus on improving social support and self-esteem and confidence of adolescent women. Early detection and treatment by heath workers assisted by community based depression screening services are essential and should be integrated in the care of adolescent pregnant and postpartum women [73].

Adolescent women were 57% less likely to undergo cesarean section and 56% less odds to undergo assisted vaginal delivery than adult women. A systematic review of published studies also showed a lower rate of cesarean delivery among young pregnant women than the general population [74]. Studies conducted in Finland [75], Romania [76], Taiwan [77], Latin America [78], and the United States of America (USA) [79] also showed a lower risk of cesarean delivery among adolescent than adult women [75,76]. Yet another study conducted in Turkey also showed similar finding [80].

The difference in the rate of cesarean section among adolescent compare to adults could be attributed to the difference in the nutrition, weight gain in pregnancy, and care received during pregnancy. Maternal undernourishment is associated with low-birth-weight babies resulting in easier vaginal delivery. Most adolescent pregnancies are unplanned, and do not receive proper attention [81]. As a result, adult women are more likely to have better nutrition and gain more weight than adolescent women. Moreover, adolescent women also have lower economic status, are uneducated, and unemployed compared with adult women, and these may affect their nutrition leading to inappropriate weight gain in pregnancy [81]. The low weight gain of adolescent women [81], and the fact that babies born to adolescent women are more likely to have low-birth weight than adult women [82–84] which may reduce the likelihood of cephalopelvic disproportion (CPD) and cesarean section than adult women. On the contrary, overweight women are at higher risk of CPD and therefore requires more caesarean delivery on account of fetal macrosomia [81]. However, where adolescents received appropriate antenatal care and adequate nutrition, the growing fetus is likely to achieve full growth potential thus requiring operative deliveries. This may explain the conflicting report of caesarean section in studies from different regions of sub-Saharan Africa [15,85].

This study found a non-significant difference in the incidence of postpartum hemorrhage among adolescent and adult women. The finding is similar with a study conducted in Nepal [86]. However, it is different from previous studies conducted in Nigeria [15] and Latin America [78], which showed a significantly higher percentage of PPH among adolescent than adult women. On the other hand, a study conducted in Thailand showed lower PPH among adolescent than adult women [87]. This could be attributed to the difference in the study period, sample size, standard of available medical services and age-related references in these studies. Moreover, recent improvement in infant and childhood nutrition that results in earlier physical maturity of adolescents may enable them to perform like adults in pregnancy and labour [86]. However, improved medical services along with active management of labour recommended by WHO is likely to revent death from PPH [88–90].

The current study found a non-significant difference in the proportion of preeclampsia among adolescent than adult women (4.5% vs. 3.8%). However, previous studies conducted in Nigeria [15,91], Cameroon [16], India [92], and Brazil [17] showed a higher risk of pregnancy induced hypertension (PIH) among adolescent than adult women. Whereas, a multi country study carried out by WHO found a lower risk of pre-eclampsia among adolescent compared to adult women [84]. Similarly, studies conducted in Ontario [93], and South Glamorgan region of Wales [94], and India [95] also showed a significantly lower risk of PIH among adolescent than adult women. These controversial findings may be due to the difference in the age-related reference and failure to adjust confounding variables in the different studies. In addition, the small sample size of this study for this specific outcome maybe the reason for non-significant difference. A large population based or a systematic review and meta-analysis study that can clearly elucidate the difference in the percentage of preeclampsia among adolescent and adult women is recommended.

Adolescent pregnancy is also linked with adverse impact on the socio-economic development of a country [66,96]. A study conducted in Ghana also showed fewer years of schooling among women who had live births than those who terminated pregnancies, suggesting the need to focus on intervention that help to reduce unintended pregnancies among adolescent women by way of sex education and introduction of effective methods of contraception to the community [97,98]. Therefore, the use of effective unintended adolescent pregnancy prevention programs is essential to reduce the high rate of adolescent pregnancy in developing countries like Ethiopia. Particularly, adolescent student-centered and community-based programs to prevent adolescent pregnancy, improve use of condoms and other contraceptive methods are essential.

This study has several strengths. First, the study used primary data collected from adolescent and adult women, which helped to obtain several sociodemographic, economic and obstetric related variables. This helped to adjust for several sociodemographic, economic, reproductive, obstetric and other comorbid conditions that could influence the adverse maternal outcomes of adolescent pregnancy. Second, the use of standardized and validated data collection tools, for example: EPDS, helped to measure some of the maternal outcomes accurately.

However, the study may be underpowered, which may be the reason for the non-significant differences in some of the adverse outcomes among adolescent and adult women. Therefore, large scale follow-up studies are recommended to identify the association of some of the adverse outcomes like preeclampsia and PPH between adolescent and adult women.

## Conclusions

Adolescent pregnancy is associated with higher proportion of adverse maternal outcomes like PPD. However, adolescent women are less likely to undergo cesarean section and assisted vaginal delivery than adult women. The proportion of gender-based violence was also significantly higher among adolescent than adult women. Therefore, health care workers need to continue treating adolescent pregnant women as a risky group for PPD and should provide necessary preventive services to reduce such risks and complications. In particular, psychosocial counselling and early diagnosis and treatment services to prevent and treat early pregnancy and childbirth complications are essential. The use of health extension workers in creating community-based awareness programs on early initiation of antenatal care and prevention of unintended adolescent pregnancy is essential. Interventions essential for prevention of GBV are also essential. Moreover, future large-scale studies are recommended to clearly elucidate the association of adolescent pregnancy with some of the adverse maternal outcomes.

## Supporting information

S1 File Data collection questionnaire in English language. (PDF)

S2 File Data collection questionnaire in Amharic language. (PDF)
