# Supplementary material for: Adverse maternal outcomes of adolescent pregnancy in Northwest Ethiopia: A prospective cohort study
Source: PLoS One. 2021 Sep 22;16(9):e0257485. doi: 10.1371/journal.pone.0257485 (PMC8457495; doi:10.1371/journal.pone.0257485)
Supplement: S1 File — (PDF) [file pone.0257485.s001.pdf]

Questionnaire No./CODE	

DATE OF INTERVIEW:	Time Interview Started: Hour: _____ Minute: _____
INTERVIEWER NAME:	Time Interview Ended: Hour: _____ Minute: _____
NAME OF HEALTH FACILITY:	

Part 1. Address		
101.	What is the name of the district you are living?	District_____________
102.	What is the urban/rural category of your kebele	1. Urban<br>2. Rural
103.	What is the name of your Kebele and local area?	1. Kebele__________________<br>2. Local area ___________________
104.	Code for name of the mother	

Part 2. Socio-demographic characteristics			
**S.No.**	**Variables**	**Response**	Skip to
201.	In what month and year were you born?<br>(write in Ethiopian calendar)	Month……………………… ☐☐<br>Don't know month………..<br>Year ………………… ☐☐☐☐<br>Don't know year…………………	
202.	How old were you at your last birthday? (Compare and correct 101 and/or 102 if inconsistent)	Age in completed years ……… ☐☐	
203.	Have you ever attended school?	1. Yes<br>2. No ⟹	**206**
204.	What is the highest level of school you attended?	1. Primary (grade 1-8)<br>2. Secondary (grade 9-12)<br>3. Technical/vocational<br>4. Higher	
205.	What is your ethnicity	1. Amhara<br>2. Tigray<br>3. Oromo<br>4. Others (specify) __________________	
206.	What is your religion	1. Orthodox<br>2. Catholic<br>3. Protestant<br>4. Muslim<br>5. Others (specify) __________________	
207.	Your father's education level	1. Unable to read and write<br>2. Can read and write<br>3. Grade1-6<br>4. Grade 7-12<br>5. Collage level and above	
208.	Your mother's education level	1. Unable to read and write<br>2. Can read and write	

		3. Grade1-6<br>4. Grade 7-12<br>5. Collage level and above	
209.	Your father's occupation	1. Daily laborer<br>2. Farmer<br>3. Civil servant<br>4. Employed in private sector<br>5. Has private business<br>6. Others (specify) ___________________	
210.	Your mother's occupation	1. Daily laborer<br>2. Farmer<br>3. Civil servant<br>4. Employed in private sector<br>5. Has private business<br>6. Others (specify) ___________________	
211.	What is your usual occupation, that is, what kinds of work do you mainly do?	1. In school<br>2. Working, (specify your work) ______________<br>3. Neither in school or working<br>4. Other(specify) ___________________	
212.	Are you currently married or living together with a man as if married?	1. Currently married ⟹<br>2. Yes, living with a man ⟹<br>3. No, not in union	216<br>216
213.	If your answer to question 212 is no, have you ever been married or lived together with a man as if married?	1. Yes, formerly married<br>2. Yes, lived with a man<br>3. No	
214.	If your answer to question 213 is no, what is your marital status now: are you widowed, divorced, or separated?	1. Widowed<br>2. Divorced<br>3. Separated	
215.	Is your husband/partner living with you now or is he staying elsewhere?	1. Living with me<br>2. Staying elsewhere	
216.	If married, your husband's occupation	1. Daily laborer<br>2. Farmer<br>3. Civil servant<br>4. Employed in private sector<br>5. Has private business<br>6. Others (specify) ___________________	
217.	Living arrangement, with whom are you living now?	1. With husband/spouse<br>2. Father and mother<br>3. With relatives<br>4. Friends<br>5. Alone<br>6. Other, specify ____________________	

Part 3. Questions related to household assets			
**S.No.**	**Variables**	**Response**	Skip to
301.	What is the main material of the floor?	1. Earth/sand<br>2. Dung<br>3. Wood planks<br>4. Palm/bamboo<br>5. Ceramic tiles<br>6. Cement<br>7. Carpet<br>8. Other (specify)________________________	
302.	Main roof material of your house	1. Corrugated iron<br>2. Thatch<br>3. Cement/concrete<br>4. Plastic sheet<br>5. Other (specify)__________________________	
303.	What is the main source of drinking water for members of your household?	1. Piped water<br>2. Water from well<br>3. Surface water<br>4. Other (specify)______________________	
304.	What kind of toilet facilities do you have in your home?	1. Flush toilet<br>2. Pit toilet/latrine<br>3. No facility/bush/field<br>4. Other (specify)______________________	
305.	What type of fuel do you mainly use for cooking in your household?	1. Electricity<br>2. Firewood, straw<br>3. Charcoal<br>4. Biogas<br>5. Kerosene<br>6. Other (specify)______________________	
306.	Does your household have electricity?	1. Yes<br>2. No	
307.	Own the house living in?	1. Yes<br>2. No	
308.	Does your household own:<br>1. A landline telephone?<br>2. A refrigerator?<br>3. A radio that is in working order?<br>4. A television that is in working order?<br>5. A bed with cotton/sponge/spring mattress?<br>6. Farm land<br>7. Have cattle<br>How many cattle?<br>8. Have horse/ donkey<br>How many?<br>9. Have sheep/goats?<br>How many?	**YES** **NO**<br>............1.......... 2<br>............1.......... 2<br>............1.......... 2<br>............1.......... 2<br>............1.......... 2<br>............1.......... 2<br>............1.......... 2<br>………………..<br>............1.......... 2<br>………………..<br>............1.......... 2<br>………………..	

309.	Does any member of your household own:<br>1. A cell phone?<br>2. A bicycle?<br>3. A motorcycle or motor scooter?<br>4. A car or truck?<br>5. An animal-drawn cart?	**YES** **NO**<br>…………1………. 2<br>…………1………. 2<br>…………1………. 2<br>…………1………. 2<br>…………1………. 2	
310.	How many rooms in your house are used for sleeping?	___________________ (write in number)	
311.	Total number of family members living in your household?	_________________ (write in number)	
312.	How much is your household monthly income (write in Ethiopian birr (ETB))	________________ ETB	

**Part 4. Past obstetric history of respondents**			
**S.No**	**Variables**	**Response**	**Skip to**
401.	What was the age at which you married for the first time?	_________________ years old	
402.	Have you ever been pregnant, other than this pregnancy	1. Yes, _________ times<br>2. No ⟹	410
403.	What was your age at your first pregnancy	________ years old	
404.	Have you ever aborted?	1. Yes, _______ times<br>2. No	
405.	Have you given birth to a child?	1. Yes, ______ children<br>2. No	
406.	How many live births did you have?	___________ (write in number)	
407.	Did you have stillbirths?	1. Yes, _________ times<br>2. No	
408.	Have you experienced an infant death?	1. Yes, _________ times<br>2. No	
409.	How many home deliveries did you have	__________(write in number)	
410.	How many institutional deliveries did you have	__________(write in number)	
411.	Thinking back to just before you got pregnant for the current pregnancy, how did you feel about becoming pregnant?	1. I wanted to be pregnant sooner<br>2. I wanted to be pregnant later<br>3. I wanted to be pregnant then<br>4. I didn't want to be pregnant then or at any time in the future	
412.	When you got pregnant with your current pregnancy, were you or your husband or partner doing anything to keep from getting pregnant? (ex. using birth control methods.)	1. Yes<br>2. No ⟹	414
413.	If yes to question number 211, Which of the following methods were you/your husband using before you got pregnant?	1. Pills<br>2. Injectables<br>3. IUCD<br>4. Implants	

		5. Condoms<br>6. Rythum method<br>7. Other (specify) ____________________	
414.	(Now I have some questions about the future.) After the child you are expecting now, would you like to have another child, or would you prefer not to have any more children?	1. Have a/another children<br>2. No more<br>3. Undecided/don't know<br>4. Other (specify) ___________________	
415.	After the child you are expecting now, how long would you like to wait before the birth of another child?	1. Years ______________________<br>2. Months _____________________<br>3. Soon/now<br>4. Don't know	
416.	Did you attend pregnancy checkups/ANC for the current pregnancy?	1. Yes, _________ times<br>2. No ⟹	420
417.	How many weeks or months pregnant were you when you first received antenatal care for the pregnancy?	Number of months |____| |____| Weeks ______	
418.	How many times did you receive antenatal care during the current pregnancy?	Number |____| |____| Don't know--------------------- 99	
419.	Where did you receive antenatal care for the current pregnancy?	1. Hospital<br>2. Health center<br>3. Private hospital/clinic<br>4. Other (Specify) __________________	
420.	During this pregnancy, were you given an injection in the arm to prevent the baby from getting tetanus, that is, convulsions after birth?	Yes<br>No	
421.	During this pregnancy, how many times did you receive tetanus injection?	______________ times	
422.	Have you received Iron and folic acid supplementation?	1. Yes, for _________ months duration<br>2. No	
423.	How far is it from your residential home to the nearby health facility (health center, hospital)?	Distance in kms_________<br>Do not know.........................99	
424.	How long does it take to walk from here to the nearby health facility (health center, hospital)?	Minutes.................................<br>Don't know minutes....................99	
425.	Previous history of hypertension	1. Yes<br>2. No	
426.	Family history of hypertension	1. Yes<br>2. No	
427.	Family history of diabetes mellitus	1. Yes<br>2. No	
428.	Do you have any other family related health condition	1. Yes, specify ______________________<br>2. No	
429.	Did you had malaria attack during the current pregnancy?	1. Yes<br>2. No	
430.	Did you have any of the following problems during the current pregnancy?<br>1. Vaginal bleeding	**Yes** **No**<br>1 2	

	2. Severe headache<br>3. Severe nausea, vomiting, or dehydration<br>4. High blood pressure, hypertension<br>5. Fever<br>6. Abdominal pain<br>7. Other ________________________	1 2<br>1 2<br>1 2<br>1 2<br>1 2<br>1 2	
431.	What have you done to alleviate the problem you had?	1. I was not sick<br>2. Nothing<br>3. Treated by health professional<br>4. Took traditional medicine<br>5. Other, specify ________________	
432.	During any of your prenatal care visits did a doctor, nurse, or other health care worker talk with you about any of the things listed below?<br>1. How drinking alcohol or smoking cigarettes during pregnancy could affect your baby<br>2. The signs and symptoms of preterm labor (labor more than 3 weeks before the baby is due)<br>3. Signs of pregnancy Complications?<br>4. What to do if your labor starts early<br>5. What to do for Pregnancy complication<br>6. Getting tested for HIV (the virus that causes AIDS)<br>7. Where to go for delivery<br>8. Post-natal period (for instance breastfeeding, nutrition, care for the child)?<br>9. Possible mood changes in the postnatal period?	**Yes** **No**<br>1 2<br>1 2<br>1 2<br>1 2<br>1 2<br>1 2<br>1 2<br>1 2<br>1 2	
433.	If you didn't attend, what was the reason?	1. I don't know there is such a service<br>2. The health unit is far away from home<br>3. I don't have any health problem<br>4. I don't like the way the health professionals handle clients<br>5. My belief doesn't allow me<br>6. Other, specify _______________	
434.	During your current pregnancy, did the healthcare providers told you to bring your spouse's to prenatal care?	1. Yes<br>2. No	
435.	Did your spouse/partner came to health facility for antenatal care purpose during the current pregnancy?	1. Yes<br>2. No	
436.	At any time during your current pregnancy, did your husband/partner push, hit, slap, kick, choke, or physically hurt you in any other way?	1. Yes<br>2. No	
437.	At any time during your current pregnancy, did your husband/partner physically forced to do sexual intercourse, had sexual intercourse when you do not want?	1. Yes<br>2. No	
438.	At any time during your current pregnancy, did your husband/partner insulted, Humiliated, done something to scare you, or threatened to hurt you?	1. Yes<br>2. No	

439.	History of hospital admission during current pregnancy	1. Yes<br>2. No	
440.	If yes to question 334, what was the reason for hospital/health center admission?	______________________________	
441.	Were you admitted to a maternity waiting homes during the current pregnancy before childbirth?	1. Yes<br>2. No	
442.	If your answer to question number 440 is yes, for how many days were you admitted to maternity waiting homes before childbirth?	________ days<br>________ hours	

Part 5. Questions related to substance abuse			
Now I am going to ask you some questions about Substance Use			
S.No.	Question	Response	Skip
501.	Did you ever drink alcoholic beverages? Like (Tela, Teji, areki or Beer).	1. Never ⟹<br>2. Drink daily<br>3. Once or twice a week<br>4. Others (specify)_______<br>5. No response	503
502.	If yes to Qn 401, did you drink alcoholic beverages during the current pregnancy? Like (Tela, Teji, areki or Beer).	1. Yes<br>2. No	
503.	Did you ever smoke tobacco products, such as cigarettes?	1. Never ⟹<br>2. Smoke daily<br>3. Once or twice a week<br>4. Others (specify)_______<br>5. No response	505
504.	If yes to Qn 403, Did you smoke tobacco products, such as cigarettes during the current pregnancy?	1. Yes<br>2. No	
505.	Have you ever chew *Khat*?	1. Never ⟹<br>2. Chew daily<br>3. Once or twice a week<br>4. Others (specify)_______<br>5. No response	507
506.	If yes to Qn 405, did you chew *Khat* during the current pregnancy?	1. Yes<br>2. No	
507.	Have you ever used other drugs such as hashish/Shisha?	1. Never ⟹<br>2. Use daily<br>3. Once or twice a week<br>4. Others (specify)_______<br>5. No response	601

508.	If yes to Qn 407, did you use these (hashish/Shisha) during the current pregnancy?	1. Yes<br>2. No	
**Part 6. Maternal outcomes during labor and delivery**			
**S.No**	**Variables**	**Response**	Skip to
601.	Is the mother delivered at this health center or referred to higher health facility?	1. Delivered at this facility ⟹<br>2. Referred to higher health facility	604
602.	If the mother is referred what is the name of the health facility the mother is referred to?	______________________________	
603.	If the mother is referred to higher health facility, what was the indication for referral? (more than one answer is possible)	1. Prolonged labour<br>2. Fetal distress<br>3. Previous cesarean section scar<br>4. Preclapsia / eclampsia<br>5. Previous fistula history<br>6. Other(specify): ________________	
604.	What was the delivery type/mode of delivery?<br>(More than one option is possible)	1. Spontaneous vaginal delivery/normal birth<br>2. Caesarean Section<br>3. Forceps delivery<br>4. Vacuum extraction<br>5. Other procedures __________________	
605.	Did the woman undergo Episiotomy?	1. Yes<br>2. No	
606.	If delivery was by Caesarean Section, what was the reason for operative delivery<br>(More than one response is possible)	1. Breech presentation<br>2. Failure to progress in labor<br>3. Failure to deliver<br>4. Fetal malposition<br>5. Non-reassuring fetal heart rate<br>6. Placenta previa<br>7. Other, specify ________________	
607.	If delivery was by Caesarean Section (C/S), what was the reason the type of caesarean section?	1. Lower uterine segment transverse C/S<br>2. Classical C/S<br>3. Inverted T C/S<br>4. Other, specify ________________	
608.	What was the presentation of the fetus during delivery?	1. Cephalic prsentation<br>2. Breech presentation<br>3. Shoulder presentation<br>4. Face presentation<br>5. Other(specify) ____________	
609.	What was the position of the fetus during delivery?	1. Ocipito anterior position<br>2. Ocipito posterior position<br>3. Right ocipito lateral position<br>4. Left ocipito lateral position<br>5. Other (specify) ______________	
610.	Gestational age of the pregnant mother during labor/delivery?	|____| |____| weeks of gestation	
611.	Time duration from initiation of labor to full cervical dilatation	________ hours	
612.	Did the mother undergo obstetric Induction of labour?	1. Yes	

		2. No	
613.	Did the mother had premature rupture of membrane (PROM)?	1. Yes<br>2. No	
614.	Who assisted the delivery?<br>(more than one answer is possible)	1. Doctor<br>2. Midwife<br>3. Nurse<br>4. Health officer<br>5. Obstetrics and gynecology specialist<br>6. Other, specify ___________	
615.	Does the mother have any of the following complications?<br>1. Preeclampsia ....<br>2. Eclampsia ....<br>3. Anteparum hemorrhage (APH) ....<br>4. Postpartum hemorrgae (PPH) ....<br>5. Shock...<br>6. Other obstetric complications (specify)___________..	**Yes (1)** **No (2)**<br>....... 1 .... 2<br>....... 1 .... 2<br>....... 1 .... 2<br>....... 1 .... 2<br>....... 1 .... 2<br>....... 1 2	
616.	If the woman has preeclampsia/eclampsia, was there proteinuria (protein in the urine)?	1. Proteinuria present<br>2. Proteinuria absent ⟹	**618**
617.	If protein urea present, what was the urine protein measurement? (write the lab result in the space provided)	______________________	
618.	What is the hemoglobin level of the mother during labor and delivery?	_____________ (write the result in g/dl)	
619.	Maternal preventive services<br>a) Vitamin A given to the mother…………………………...<br>b) HIV testing accepted ………………………………………<br>c) HIV test result………………………………………………	**Yes (1)** **No (2)**<br>……. 1 …. 2<br>……. 1 …. 2.<br>1. Positive 2. Negative	
620.	What is the maternal status after delivery?	1. Stable<br>2. Unstable/ deteriorated<br>3. Died ⟹	**621**
621.	If the mother is not alive, what was the main cause of death diagnosed?	1. Post-partum hemorrhage (PPH)<br>2. Hypertension disorder (Specify): ___________<br>3. Infectious disease diagnosis (Specify): _______<br>4. Other(Specify)__________________	

**Part 7. Newborn Outcomes**			
701.	What is the sex of the newborn baby?	1. Male<br>2. Female	
702.	What was the newborn outcome	1. Alive<br>2. Dead ⟹	717
703.	What was the birthweight of the baby (in grams)?	_____________________ grams	
704.	APGAR score 1st minute after birth	__________________ (write the score)	
705.	APGAR score 5th minute after birth	__________________ (write the score)	
706.	New born preventive services<br>a) BCG given to the newborn……………………….......<br>b) OPV 0 given to the newborn……………………….....	**Yes (1)** **No (2)**<br>……. 1 …. 2<br>……. 1 …. 2.	
707.	Any evidence of jaundice in the new born baby in first 24 hours of life, eg. yellow palms and soles at any age.	1. Yes<br>2. No	
708.	Does the newborn has any form of congenital malformation?	1. Yes<br>2. No ⟹	710

709.	If the newborn has congenital malformation, what was the type/diagnosis of malformation?	____________________________________	
710.	Does the newborn baby have any of the following problems?<br>a) Prematurity ……………………………………………<br>b) Sepsis……………………………………………………<br>c) Respiratory distress ……………………………………<br>d) Perinatal asphyxia………………………………………<br>e) Anemia…………………………………………………<br>f) Congenital malformation …………………………………<br>g) Meconium aspiration ……………………………………<br>h) Other (specify) ____________________………………………	**Yes (1)** **No (2)**<br>……. 1 …. 2<br>……. 1 …. 2<br>……. 1 …. 2<br>……. 1 …. 2<br>……. 1 …. 2<br>……. 1 …. 2<br>……. 1 …. 2<br>……. 1 …. 2	
711.	Treatment given to the newborn baby<br>a) Oxygen/ resuscitation…………………………………<br>b) Kangaroo mother care (KMC) ………………………<br>c) Antibiotics ……………………………………………<br>d) Glucose…………………………………………………<br>e) Blood transfusion………………………………………<br>f) Other (specify) ___________________……………………	**Yes (1)** **No (2)**<br>……. 1 …. 2<br>……. 1 …. 2<br>……. 1 …. 2<br>……. 1 …. 2<br>……. 1 …. 2<br>……. 1 …. 2	
712.	If treatment is given to the newborn baby, what was the treatment outcome?<br>a) Improved……………………………………………<br>b) No change…………………………………………<br>c) Died…………………………………………………<br>d) Referred………………………………………………	**Yes (1)** **No (2)**<br>……. 1 …. 2<br>……. 1 …. 2<br>……. 1 …. 2<br>……. 1 …. 2	
713.	Did the mother identified on the type of breast feeding?	1. Yes<br>2. Not at all	
714.	If mother starts breast feeding, within how many hours after childbirth did she start breastfeeding?	____________ hours after childbirth	
715.	Was the newborn admitted to neonatal intensive care unit (ICU)?	1. Yes<br>2. No	
716.	If yes to question 615, what was the reason for the neonatal ICU admission?	a) Prematurity<br>b) Infection<br>c) Asphyxia<br>d) Congenital malformation<br>e) Other (specify) ____________________	
717.	If the baby died, what was the cause of death?<br>1. Prematurity ……………………………………………<br>2. Infection ………………………………………………<br>3. Asphyxia ………………………………………………<br>4. Congenital malformation ………………………………<br>5. Other (specify) ___________________……………………	**Yes (1)** **No (2)**<br>……. 1 …. 2<br>……. 1 …. 2<br>……. 1 …. 2<br>……. 1 …. 2<br>……. 1 …. 2	
718.	If dead, what was the type?	1. Still birth<br>2. Death of the baby in the health facility after live birth	

Part 8. Data collection before discharge from the health facility			
**S.No**	**Variables**	**Response**	Skip to
801.	Does the mother has Signs and symptoms of post partum hemorrhage?<br>1. Sudden and profuse blood loss or persistent increase blood loss.............<br>2. Faintness...<br>3. Dizziness ....<br>4. Palpitations/tachycardia...<br>5. Other (specify) ____________________.......................................	**Yes (1)** **No (2)**<br>....... 1 .... 2<br>....... 1 .... 2<br>....... 1 .... 2<br>....... 1 .... 2<br>....... 1 2	
802.	Signs and symptoms of pre-eclampsia/eclampsia (after birth)?<br>a) Headaches...<br>b) Visual disturbances..<br>c) Nausea...<br>d) Vomiting...<br>e) Epigastric or hypochondrial pain..<br>f) Feeling faint..<br>g) Convulsions...<br>h) Other (specify) _________________.....................	**Yes (1)** **No (2)**<br>....... 1 .... 2<br>....... 1 .... 2<br>....... 1 .... 2<br>....... 1 .... 2<br>....... 1 .... 2<br>....... 1 .... 2<br>....... 1 .... 2<br>....... 1 2	
803.	Does the mother have any signs and symptoms of infection?<br>a) Fever...<br>b) Shivering...<br>c) Abdominal pain..<br>d) Offensive vaginal discharge..<br>e) Other (specify) _________________...............................	**Yes (1)** **No (2)**<br>....... 1 .... 2<br>....... 1 .... 2<br>....... 1 .... 2<br>....... 1 .... 2<br>....... 1 2	
804.	Duration of stay in health facility after birth before discharge to home? (write the answer in hours)	_____ hours	

Data collection at six weeks postpartum period

Edinburgh Postnatal Depression Scale (EPDS) tool used to assess postnatal depression

As you have recently had a baby, we would like to know how you are feeling. Please UNDERLINE which comes closest to how you have felt IN THE PAST 7 DAYS, not just how you feel today.

**S.No**	**Questions**	**Responses**
901.	In the Past 7 Days: I have been able to laugh and see the funny side of things as much as I always could.	0– As much as I always could<br>1– Not quite so much now.<br>2– Definitely not so much now<br>3 – Not at all
902.	In the Past 7 Days: I have looked forward with enjoyment to things.	0 – As much as I ever did<br>1 – Rather less than I used to<br>2 – Definitely less than I used to<br>3– Hardly at all
903.	In the Past 7 Days: I have blamed myself unnecessarily when things went wrong.	3– Yes, most of the time.<br>2– Yes, some of the time<br>1– Not very often

		0– No, never
904.	In the Past 7 Days: I have been anxious or worried for no good reasons.	0– No, not at all.<br>1- Hardly, ever<br>2– Yes, sometimes<br>3 - Yes, very often
905.	In the Past 7 Days: I have felt scared or panicky for no very good reason.	3– Yes, quite a lot<br>2 – Yes, sometimes<br>1 – No, not much<br>0 – No, not at all
906.	In the Past 7 Days: Things have been getting on top of me.	3– Yes, most of the time I haven't been able to cope at all<br>2 - Yes, sometimes I haven't been coping as well as usual<br>1 – No, most of the time I have coped quite will<br>0 – No, I have been coping as well as ever
907.	In the Past 7 Days: I have been so unhappy that I have had difficulty sleeping	3– Yes, most of the time<br>2 – Yes, sometimes<br>1 – Not very often<br>0 – No, not at all
908.	In the Past 7 Days: I have felt sad or miserable	3-Yes, most of the time<br>2- Yes, quite often<br>1- Not very often<br>0- No, not at all
909.	In the Past 7 Days: I have been so unhappy that I have been crying	3- Yes, most of the time<br>2- Yes, quite often<br>1 - Only occasionally<br>0 – No, not at all
910.	In the Past 7 Days: The thought of harming myself has occurred to me.	3-Yes, quite often<br>2-Sometimes<br>1-Hardly ever<br>0-Never

Thank you very much for taking the time to answer!

(For data collectors: After you fill the above tool on postnatal depression (part 10) please add the questions from 901-910 and write the result here. **Total: ____/ 30 .** If the total result is 10 or above, please refer the woman to the nearest health facility for further follow-up and treatment purpose.
