# Supplementary material for: Adverse maternal outcomes of adolescent pregnancy in Northwest Ethiopia: A prospective cohort study
Source: PLoS One. 2021 Sep 22;16(9):e0257485. doi: 10.1371/journal.pone.0257485 (PMC8457495; doi:10.1371/journal.pone.0257485)
Supplement: S2 File — (PDF) [file pone.0257485.s002.pdf]

Questionnaire No./CODE	RH-

DATE OF INTERVIEW:	Time Interview Started: Hour: _____ Minute: _____
INTERVIEWER NAME:	Time Interview Ended: Hour: _____ Minute: _____
NAME OF HEALTH FACILITY:	

ክፍል 1. የተጠያቂዋ አድራሻ		
101.	የምትኖሪበት የወረዳ ስም ማን ይባላል?	ወረዳ ስም _______________
102.	የምትኖሪበት ቀበሌ የከተማ / የገጠር ክፍል ነው?	1. ከተማ 2. ገጠር
103.	የምትኖሪበት የቀበሌ እና የጎጥ ስም ማን ይባላል?	1. ቀበሌ ___________________<br>2. ጎጥ_________________________________
104.	የእናት ስም ኮድ	

ክፍል 2. የተጠያቂ ማህበራዊ ባህሪያት በተመለከተ የሚጠየቁ ጥያቄዎች			
ተ.ቁ	ጥያቄዎች	መልስ	Skip to
201.	በየትኛው ወር እና ዓመት ተወለዱ?<br>(በኢትዮጵያ የቀን መቁጠሪያ/ ዓመተ ምህረት ይጻፉ)	ወር.............................>> ☐☐<br>ወሩን አላውቀውም............<br>ዓመት ....>> ☐☐☐☐<br>ዓመቱን አላውቀውም.....................	
202.	ባለፈው የልደት ቀንዎ እድሜዎት ምን ያህል ነበር?<br>(ጥያቄ 201 እና 202 ካልተመሳሰለ አስተካክል/ድጋሚ ጠይቅ/ቁ)	ዕድሜ .... ዓመት ☐☐	
203.	ትምህርት ተከታትለዋል?	1. አዎ<br>2. የለም →	206
204.	እርስዎ የተማሩበት ከፍተኛው የትምህርት ቤት ደረጃ ምን ያህል ነው?	1. አንደኛ ደረጃ (ከ1-8ኛ ክፍል<br>2. ሁለተኛ ደረጃ (ከ9-12ኛ ክፍል)<br>3. ቴክኒክ እና ሞያ<br>4. ከፍተኛ ትምህርት	
205.	የእርስዎ ጎሳ /ብሄር ምንድነው	1. አማራ<br>2. ትግራይ<br>3. ኦሮሞ<br>4. ሌሎች (ይግለጹ)__________	
206.	ሃይማኖትሽ ምንድን ነው	1. ኦርቶዶክስ<br>2. ካቶሊክ<br>3. ፕሮቴስታንት<br>4. ሙስሊም<br>5. ሌሎቹ (ይግለጹ) ____________________	
207.	የአባትሽ የትምህርት ደረጃ	1. ማንበብና መጻፍ አይችልም<br>2. ማንበብና መጻፍ ይችላል<br>3. ከ 1-6 ክፍል<br>4. ከ 7 ኛ እስከ 12 ኛ<br>5. የኮሌጅ ደረጃ እና በላይ	
208.	የእናትሽ የትምህርት ደረጃ	1. ማንበብና መጻፍ አትችልም<br>2. ማንበብና መጻፍ ትችላለች<br>3. ከ 1-6 ክፍል<br>4. ከ 7 ኛ እስከ 12 ኛ<br>5. የኮሌጅ ደረጃ እና በላይ	
209.	የአባትሽ ሥራ	1. የቀን ሰራተኛ	

		2. ገበሬ<br>3. የመንግስት ሰራተኛ<br>4. በግሉ ዘርፍ ውስጥ ተቀጥሮ ይሠራል<br>5. የግል ንግድ አለው<br>6. ሌሎች (ይግለጹ)________	
210.	የእናትሽ ሥራ	1. የቀን ሰራተኛ<br>2. ገበሬ<br>3. የመንግስት ሰራተኛ<br>4. በግሉ ዘርፍ ውስጥ ተቀጥራ ትሠራለች<br>5. የግል ንግድ አለው<br>6. ሌሎች (ይግለጹ)____________	
211.	የተለመደው ሥራሽ ምንድን ነው? (ምን ዓይነት ሥራዎችን በዋነኝነት ያከናውናሉ?)	1. በትምህርት ቤት<br>2. በስራ (ስራዎን ይግለጹ) __________<br>3. በትምህርት ቤት ወይም በሥራ ላይ አይደለም<br>4. ሌላ (ይግለጹ) _____________	
212.	በአሁኑ ጊዜ ባለትዳር ነሽ?<br><br>(ካልሆንሽ ከጋብቻ ውጭ ከወንዶች ጋር እየኖርሽ ነው?)	1. በአሁኑ ጊዜ የተጋባሁ ነኝ<br>2. ትዳር አልያዝኩም፣ ነገር ግን ከወንድ ጋር እየኖርሁ ነው<br>3. አልተጋባሁ፣ ብቻዬን ነው የምኖረው	216<br>216
213.	ለጥያቄ 212 መልስዎ አልተጋባሁም ከሆነ, ካሁን በፊት ትዳር ይዘው ወይም ከወንድ ጋር አብረው ኖረው ያውቃሉ?	1. አዎ, ቀደም ሲል አግብቻለሁ<br>2. አዎ ከወንድ/ዶች ጋር እኖር ነበር<br>3. አይ፣ አግብቼ አላውቅም ወይንም ከወንድ ጋር አብሬ አልኖርኩም	
214.	ለጥያቄ ቁጥር 213 መልስዎ “አይ አግብቼ አላውቅም” ከሆነ የጋብቻ ሁኔታዎ አሁን ምን ይመስላል?	1. ባለቤቴ ሞቶብኛል<br>2. የተፋታሁ ነኝ<br>3. የተለያየሁ ነኝ	
215.	ባለቤትሽ ወይም ጓደኛሽ አሁን ከአንቺ ጋር ይኖራል? ወይስ ሌላ ቦታ ነው ያለው?	1. ከእኔ ጋር ነው የሚኖረው<br>2. 2. ሌላ ቦታ ነው የሚኖረው	
216.	ካገባሽ፣ የባለቤትሽ ሥራ ምንድን ነው?	1. የቀን ሰራተኛ<br>2. ገበሬ<br>3. የመንግስት ሰራተኛ<br>4. በግሉ ዘርፍ ውስጥ ተቀጥሬ እሠራለሁ<br>5. የግል ንግድ አለኝ<br>6. ሌሎች (ይግለጹ)______________	
217.	አሁን ከማን ጋር ነው የምትኖሪው?	1. ከባለቤት/ ከትዳር ጓደኛ ጋር<br>2. አባትና እናቶች<br>3. ከዘመዶች<br>4. ጓደኞች<br>5. ብቻዬን<br>6. ሌላ, (ይግለጹ)___________	

ክፍል 3. ስለመኖርያ ቤት/ቤተሰብ የሚመለከቱ መጠይቆች፣ አሁን ስለቤተሰብዎ እና ስለሚኖሩበት ቤት አንዳንድ ጥያቄዎችን እጠይቃለሁ።

ተ.ቁ	ጥያቄዎች	መልስ	Skip to
301.	የምትኖሩበት ቤት ወለል ከምንድን ነው የተሰራው?	1. ከአፈር/አሸዋ<br>2. በእበት የተለቀለቀ<br>3. ከእንጨት<br>4. ከሸንበቆ/ቀርቅሃ<br>5. ከሴራሚክ<br>6. ከሲሚንቶ<br>7. ከምንጣፍ<br>8. ሌላ ካለ (ይገለፅ)......................	
302.	የመኖርያ ቤትዎ ጣሪያ ከምንድን ነው የተሰራው?	1. በቆርቆሮ የተሰራ<br>2. የሳር ቤት<br>3. ከሲሚንቶ<br>4. ከፕላስቲክ የተሰራ ጣርያ	

		5. ሌላ(ይገለፅ)	
303.	ለቤተሰብ አባላት የሚሆን ዋና የመጠጥ ውኃ ምንጭ ምንድን ነው?	1. የቧንቧ ውሃ<br>2. ከጥልቅ ጉድጓድ የተገኘ ውሃ<br>3. የምንጭ/የወንዝ ውሃ<br>4. ሌላ ካለ (ይገለፅ)	
304.	በቤትዎ ውስጥ ምን ዓይነት መጸዳጃ ቤት/ቶች አሉዎት?	1. በውሃ የሚሰራ ሽንት ቤት<br>2. የጉድጓድ ደረቅ ሽንት ቤት<br>3. ሽንት ቤት የለም/መስክ ላይ /ጫካ ላይ<br>4. ሌላ ካለ (ይገለፅ)__________________________	
305.	በቤተሰብዎ ውስጥ ምግብ ለማብሰል የምትጠቀሙበት ነዳጅ ምንድን ነው ?	1. የኤሌክትሪክ ኃይል<br>2. እንጨት/ ገለባ<br>3. ከሰል<br>4. ባዮጋዝ<br>5. ነጭ ጋዝ<br>6. ሌላ ካለ (ይገለፅ)	
306.	የእርስዎ ቤተሰብ/መኖሪያ ቤት የኤሌክትሪክ ኃይል አለው?	1. አዎ 2. የለም	
307.	በአሁኑ ሰዓት የሚኖሩበት ቤት የራስዎ/ነው?	1. አዎ 2. የለም	
308.	በእርስዎ ቤተሰብ/መኖሪያ ቤት የትኞቹ የቤት እቃዎች አሉ?<br>1. መደበኛ/ተንቀሳቃሽ ያልሆነ/የቤት ስልክ..........................<br>2. ማቀዝቀዣ/ፍሪጅ..<br>3. ሬዲዮ...<br>4. ቴሌቪዥን..<br>5. ከጥጥ / ስፖንጅ / የተሰራ ፍራሽ ያለው አልጋ?..................<br>6. የእርሻ መሬት አላችሁ...<br>7. ከብቶች አላችሁ...<br>ምን ያህል ከብቶች አላችሁ?..<br>8. ፈረስ / አህያ...<br>ምን ያህል ፈረስ / አህያ አላችሁ?.....................................<br>9. በግ / ፍየል አላችሁ?...<br>ምን ያህል በግ / ፍየል አላችሁ?.....................................	አዎ የለም<br>...........1.......... 2<br>...........1.......... 2<br>...........1.......... 2<br>...........1.......... 2<br>...........1.......... 2<br>...........1.......... 2<br>...........1.......... 2<br>…………..<br>...........1.......... 2<br>…………<br>...........1.......... 2<br>…………..	
309.	ከቤተሰብዎ መካከል የሚከተሉት እቃዎች ያለው የቤተሰብ አባል አለ ?<br>1. የሞባይል ስልክ?...<br>2. ብስክሌት?..<br>3. ሞተርሳይክል ወይም ሞተር...<br>4. መኪና?..<br>5. በእንስሳት የሚሳብ ጋሪ?..	አዎ የለም<br>...........1.......... 2<br>...........1.......... 2<br>...........1.......... 2<br>...........1.......... 2<br>...........1.......... 2	
310.	በቤታችሁ ውስጥ ለመተኛት/ለመኝታ ክፍል የሚሆን ምን ያህል ክፍሎች ይጠቀማሉ?	_______________የመኝታ ክፍሎች (በቁጥር ይገለፅ)	
311.	በቤትዎ ውስጥ የሚኖሩ የቤተሰብዎ ጠቅላላ ቁጥር ስንት ነው?	_________________ (በቁጥር ይገለፅ)	
312.	የእርስዎ ቤተሰብ ወርሃዊ ገቢ ምን ያህል ነው? (በኢትዮጲያ ብር ይገለፅ)	________________ ብር	
**ክፍል 4. ስለ ፅንስ እና የወሊድ ታሪክን በተመለከተ**			
**ተ.ቁ**	**ጥያቄዎች**	**መልስ**	**Skip to**
401.	ካገባሽ፣ ለመጀመሪያ ግዜ ለጋብቻ ያገባሽበት ዕድሜ ስንት ነበር?	_________________ ዓመት	
402.	ከዚህ እርግዝና ሌላ ካሁን በፊት አርግዘሽ ታውቂያለሽ?	1. አዎ, _________ ጊዜ(ቁጥር ይፃፍ)<br>2. የለም ⟹	410
403.	በመጀመሪያው የእርግዝና ጊዜ ዕድሜሽ ምን ያህል ነበር?	________________ ዓመት	
404.	አስወርዶሽ ያውቃል?	1. አዎ, _________ ጊዜ(ቁጥር ይፃፍ)<br>2. የለም	
405.	ካሁን በፊት ልጅ ወልደሽ ታውቂያለሽ?	1. አዎ, _________ ልጆች(ቁጥር ይፃፍ)<br>2. የለም	
406.	ስንት በሂወት የተወለዱ ህፃናት ቁጥር አሉሽ?	____________ (በቁጥር ይፃፉ)	
407.	ካሁን በፊት ሞተው የተወለዱ ህፃናት ነበሩሽ?	1. አዎ, _________(በቁጥር ይፃፉ)<br>2. የለም	

408.	ካሁን በፊት የሕፃን ሞት አጋጥሞሽ ያውቃል?	1. አዎ, _________(በቁጥር ይፃፉ)<br>2. የለም	
409.	ካሁን በፊት በቤት ውስጥ/ከህክምና ተቋም ውጭ ልጅ ወልደሽ ታውቂያለሽ?	1. አዎ, _________ጊዜ (በቁጥር ይፃፉ)<br>2. የለም	
410.	ካሁን በፊት በጤና ተቋም ውስጥ ልጅ ወልደሽ ታውቂያለሽ?	1. አዎ, _________ጊዜ (በቁጥር ይፃፉ)<br>2. የለም	
411.	የአሁኑን እርግዝና ከመፀነስሽ በፊት፣ ስለእርግዝናው ምን ታስቢ ነበር?	1. በጊዜው ለመፀነስ ፈልጌ ነበር<br>2. ትንሽ መቆየት እፈልግ ነበር<br>3. በጊዜው ለመፀነስ አልፈለኩም ነበር፣ አሁን ግን ፈልጌዋለሁ<br>4. በጊዜው ወይንም ከዚያ በኋላ ለመፀነስ አልፈለኩም ነበር	
412.	በአሁኑ እርግዝና ወቅት፣ አንቺ ወይም ባለቤትሽ እርግዝናን ለመከላከል ሞክራችሁ ነበር? (ለምሳሌ የወሊድ መቆጣጠርያ ዘዴዎችን በመጠቀም)	1. አዎ<br>2. የለም →	414
413.	ለጥያቄ ቁጥር 412 መልስሽ “አዎ” ከሆነ፣ ከሚከተሉት የእርግዝና ዘዴዎች መካከል የትኛውን ዘዴ ተጠቅመሻል?	1. በአፍ የሚወሰዱ የእንክብለ መድሃኒቶች<br>2. የእርግዝና መከላከያ መርፌ<br>3. በማህፀን ውስጥ የሚቀበር ሉፕ<br>4. ክንድ ላይ የሚቀበር መድሃኒት<br>5. ኮንዶም<br>6. በተፈጥሯዊ መንገድ<br>7. ሌላ፣ ይግለጹ _______________	
414.	(ስለወደፊቱ አንዳንድ ጥያቄዎች አሉኝ).<br>አሁን ከምትጠብቂው ልጅ በኋላ ሌላ ልጅ እንዲኖርሽ ትፈልጊያለሽ? ወይስ ተጨማሪ ልጅ ላለመውለድ ትመርጫለሽ?	1. ሌላ ልጅ ወይም ልጆች እፈልጋለሁ<br>2. የለም ሌላ ልጅ አልፈልግም/ይበቃኛል<br>3. አልወሰንኩም / አላውቅም<br>4. ሌላ፣ ይግለጹ _______________	
415.	አሁን የምትጠብቂውን ልጅ ከወለድሽ በኋላ፣ ሌላ ልጅ ከመወለድሽ በፊት ለምን ያህል ጊዜ መጠበቅ ትፈልጊያለሽ? (በቁጥር ይፃፍ)	1. _______________ዓመት<br>2. _______________ወራት<br>3. በቅርቡ / አሁን<br>4. አላውቅም	
416.	በአሁኑ የእርግዝና ወቅት፣ በጤና ተቋም የእርግዝና ክትትል / አድርገሻል?	1. አዎ<br>2. ክትትል አላደረኩም →	420
417.	ካሁን በፊት ክትትል አድርገሽ ከሆነ፣ ለመጀመርያ ጊዜ የእርግዝና ክትትል ስታደርጊ፣ የምን ያክል ወራት/ሳምንታት ነብሰ ጡር ነበርሽ?	ወራት |____| |____| አላውቅም ----------------- 99<br>ሳምንታት _________________	
418.	በአሁኑ የእርግዝና ወቅት፣ በጠቅላላው ምን ያክል ጊዜ የጤና ተቋም የእርግዝና ክትትል አድርገሻል?	በቁጥር |____| |____| ጊዜ<br>አላውቅም --------------------- 99	
419.	በአሁኑ የእርግዝና ወቅት፣ የቅድመ ወሊድ ክትትል ያገኘሽው ከየት ነበር?	1. ሆስፒታል<br>2. የጤና ጣቢያ ማዕከል<br>3. የግል ሆስፒታል / ክሊኒክ<br>4. ሌላ (ዝርዝር ይግለጹ)__________	
420.	በዚህ የእርግዝና ወቅት፣ የቴታነስ መከላከያ ክትባት በክንድሽ ተሰጥቶሽ ያውቃል?	1. አዎ<br>2. የለም	
421.	በዚህ የእርግዝና ወቅት የቴታነስ መከላከያ ክትባት በክንድሽ ተሰጥቶሽ የሚያውቅ ከሆነ፣ በዚህ እርግዝና ወቅት ቲታነስ መርፌ ለምን ያህል ጊዜ ነው የወሰድሽው?	______________ ጊዜ	
422.	በዚህ እርግዝና ወቅት የ” አይረን እና ፎሊክ አሲድ” (ለደም ማነስ ችግርን ለመከላከል የሚወሰድ) እንክብል መድሃኒት አግኝተሻል/ወስደሻል?	1. አዎ, ለ _________ ወራት ቆይታ<br>2. አልወሰድኩም	
423.	ከአንቺ መኖሪያ ቤት ወደ ጤና ተቋም (የጤና ጣቢያ ወይም ሆስፒታል) ለመሄድ በግምት ምን ያህል ርቀት ይሆናል?	ያለው ርቀት _________ ኪ. ሜ<br>አላውቅም 99	
424.	ከአንቺ መኖሪያ ቤት ወደ ጤና ተቋም (የጤና ጣቢያ ወይም ሆስፒታል) በእግር ጉዞ ለመሄድ ምን ያህል ጊዜ ይፈጅብሻል?	ደቂቃዎች ....<br>አላውቅም 99	
425.	ካሁን በፊት የደም ግፊት ህመም አሞሽ ያውቃል?	1. አዎ<br>2. የለም	
426.	ከቤተሰቦችሽ መካከል የደም ግፊት ህመም አሞት የሚያውቅ የቤተሰብ አባል አለ?	1. አዎ<br>2. የለም	

427.	ከቤተሰቦችሽ መካከል የስኳር በሽታ/ ህመም አሞት የሚያውቅ የቤተሰብ አባል አለ?	1. አዎ<br>2. የለም	
428.	ከቤተሰብ ወደ ቤተሰብ የሚተላለፍ በሽታ/ ተዛማጅ የጤና ሁኔታ አለሽ?	1. አዎ፣ ይገለፅ _________________<br>2. የለም	
429.	በአሁኑ የእርግዝና ወቅት፣ የወባ በሽታ አሞሽ ነበር?	1. አዎ<br>2. የለም	
430.	በአሁኑ የእርግዝና ወቅት፣ ከሚከተሉት ችግሮች መካከል የትኞቹ አጋጥሞሻል?<br>1. ከብልት የሚወጣ የደም ፈሳሽ...<br>2. ከባድ ራስ ምታት..<br>3. ከፍተኛ የማቅለሽለሽ ስሜት ወይም ማስታወክ ……………<br>4. ከፍተኛ የደም ግፊት..<br>5. ትኩሳት...<br>6. የሆድ ቁርጠት...<br>7. ሌላ (ይገለፅ)________________________.............................	**አዎ** **የለም**<br>1 2<br>1 2<br>1 2<br>1 2<br>1 2<br>1 2<br>1 2	
431.	በእርግዘዝና ወቀት ያጋጠመሽን የጤና ችግር ለመቅረፍ ምን አድርገሽ ነበር?	1. አልታመምኩም ነበር<br>2. ምንም<br>3. በጤና ባለሙያ ህክምና አግኝቻለሁ<br>4. ባህላዊ ሕክምና ተጠቅሜያለሁ<br>5. ሌላ, ይገለፅ _____________	
432.	በቅድመ ወሊድ እንክብካቤ ክትትል ወቅት ዶክተር፣ ነርስ ወይም ሌላ የጤና ባለሞያ ሠራተኛ ከዚህ በታች የተዘረዘሩትን ነገሮች አነጋግሮሻል?<br>1. በእርግዝና ወቅት አልኮል መጠጣት ወይም ሲጋራ ማጨስ ልጅሽን ሊጎዳ እንደሚችል....................................<br>2. ትክክለኛው የወሊድ ቀን ከመድረሱ በፊት ሊያጋጥም ስለሚችለው የወሊድ ምጥ/ያለጊዜው የሚመጣ ምጥ ምልክቶች...<br>3. በእርግዝና ወቅት ሊያጋጥሙ ስለሚችሉ ችግሮች እና ምልክቶቻቸው..<br>4. የወሊድ ምጥ ቀድሞ ቢመጣ ምን ማድረግ እንዳለብሽ..<br>5. በእርግዝና ወቅት ለሚያጋጥሙ ችግሮች ምን ማድረግ እንዳለብሽ...<br>6. ስለ ኤች አይ ቪ/ኤድስ ምርመራ ማድረግ አስፈላጊነት...<br>7. ልጅ ለመውለድ የት መሄድ እንዳለብሽ..<br>8. ከወሊድ በኋላ ስላለው ጊዜ (ለምሳሌ የጡት ወተት ስለማጥባት፣ ስለ አመጋገብ፣ ስለ ልጅ እንክብካቤ)? ……<br>9. ከወሊድ በኃላ ሊያጋጥም ስለሚችል የስሜት ለውጥ/ የጭንቀት ችግር?......................................	**አዎ** **የለም**<br>1 2<br>1 2<br>1 2<br>1 2<br>1 2<br>1 2<br>1 2<br>1 2<br>1 2	
433.	በአሁኑ የእርግዝና ወቅት፣ የቅድመ ወሊድ እንክብካቤ ክትትል ካላደረግሽ፣ ያላደረግሽበት ምክንያቱ ምንድን ነው?	1. እንዲህ ያለ አገልግሎት ስለመኖሩ አላውቅም<br>2. የጤና ተቋም ከምኖርበት ቤት ስለሚርቅ<br>3. ምንም ዓይነት የጤና ችግር ስላልነበረኝ<br>4. የጤና ባለሙያዎች ታካሚዎችን የሚያክሙበት/የሚይዙበትን መንገድ አልወድም<br>5. እምነቴ ስለማይፈቅድልኝ<br>6. ሌላ, ይገለፅ _________________	
434.	በአሁኑ የእርግዝና ወቅት፣ የቅድመ ወሊድ እንክብካቤ ክትትል ስታደርጊ ባለቤትሽ/ ጓደኛሽ ወደ ጤና ተቋም እንዲመጣ ተነግሮሽ ነበር?	1. አዎ<br>2. የለም	
435.	ባለቤትሽ/ጓደናሽ በእርግዝና ክትትል ወቅት አብሮሽ በህክምና ተቋም ተገኝቶ ነበር?	1. አዎ 2. የለም	
436.	በአሁኑ የእርግዝና ወቅት በማንኛውም ጊዜ የባለቤት / የትዳር ጓደኛሽ በጉልበት ገፍቶሽ, በጥፊ/በእርግጫ መትቶሽ, ድብደባ ወይም በሌላ መንገድ አካላዊ ጉዳት አድርሶብሽ ያውቃል?	1. አዎ<br>2. የለም	
437.	በአሁኑ የእርግዝና ወቅት በማንኛውም ጊዜ የባለቤት / የትዳር ጓደኛሽ በግብረ ሥጋ ግንኙነት የግድ እንድትፈጽሚ ወይም አንቺ በማትፈልጊበት ጊዜ ወሲባዊ ግንኙነት እንድትፈጽሚ አስገድዶሽ ያውቃል?	1. አዎ<br>2. የለም	
438.	በአሁኑ የእርግዝና ወቅት በማንኛውም ጊዜ፣ የባለቤት / የትዳር ጓደኛሽ ሰድቦሽ፣ አዋርዶሽ፣ ወይም አንቺን ለመጉዳት አስፈራርቶሽ ያውቃል ?	1. አዎ<br>2. የለም	
439.	በአሁኑ የእርግዝና ወቅት፣ በህመም ምክንያት ለህክምና ሆስፒታል/ጤና ተቋም ገብተሽ ነበር?	1. አዎ 2. የለም	
440.	ለጥያቄ ቁጥር 339 መልስዎ “አዎ” ከሆነ፣ ሆስፒቲል/ጤና ተቋም የገባሽበት ህመም/ ምክንያት ምን ነበር?		
441.	በአሁኑ የእርግዝና ወቅት፣ ለወሊድ ዝግጅት እንዲሆን በእናቶች የወሊድ ማቆያ ክፍል ቆይተሽ ነበር?	1. አዎ 2. የለም	
442.	ለጥያቄ ቁጥር 440 መልስሽ “አዎ” ከሆነ፣ ለምን ያክል ቀናት እና ሰዓታት በእናቶች ማቆያ ክፍል ነበርሽ?	______________ ቀናት<br>_______________ሰዓታት	

ክፍል 5. ስለ ሱስ የሚያስይዙ ነገሮች መጠቀምን የሚመለከቱ ጥያቄዎች			
አሁን ስለ ሱስ የሚያስይዙ ነገሮች አጠቃቀም አንዳንድ ጥያቄዎችን እጠይቅሻለሁ			
**ተ.ቁ**	**ጥያቄዎች**	**ምላሾች**	**Skip to**
501.	የአልኮል መጠጦችን ጠጥተሽ ታውቂያለሽ? (ለምሳሌ፣ እንደ ጠላ፣ ጠጅ፣ አረቂ ወይም ቢራ የመሳሰሉትን)	1. በጭራሽ ⟹<br>2. በየቀኑ እጠጣለሁ<br>3. በሳምንት አንዴ ወይም ሁለት ጊዜ<br>4. ሌሎች (ይግለጹ) _______<br>5. መልስ የለም	503
502.	ካሁን በፊት አልኮል ጠጥተሽ የምታውቂ ከሆነ፣ በአሁኑ የእርግዝና ወቅት የአልኮል መጠጦችን ጠጥተሽ ታውቂያለሽ? (ለምሳሌ፣ እንደ ጠላ፣ ጠጅ፣ አረቂ ወይም ቢራ የመሳሰሉትን)	1. አዎ<br>2. የለም	
503.	እንደ ሲጋራዎች ያሉ የትንባሆ ምርቶችን አጭሰሽ/ ተጠቅመሽ ታውቂያለሽ?	1. በጭራሽ ⟹<br>2. በየቀኑ እጠቀማለሁ<br>3. በሳምንት አንዴ ወይም ሁለት ጊዜ<br>4. ሌሎች (ይግለጹ) _______<br>5. መልስ የለም	505
504.	ካሁን በፊት እንደ ሲጋራዎች ያሉ የትንባሆ ምርቶችን አጭሰሽ/ ተጠቅመሽ የምታውቂ ከሆነ፣ በአሁኑ የእርግዝና ወቅት እንደ ሲጋራዎች ያሉ የትንባሆ ምርቶችን ተጠቅመሽ ታውቂያለሽ?	1. አዎ<br>2. የለም	
505.	ካሁን በፊት ጫት ቅመሽ ታውቂያለሽ?	1. በጭራሽ ⟹<br>2. በየቀኑ እቅማለሁ<br>3. በሳምንት አንዴ ወይም ሁለት ጊዜ<br>4. ሌሎች (ይግለጹ) _______<br>5. መልስ የለም	507
506.	ካሁን በፊት ጫት ቅመሽ የምታውቂ ከሆነ፣ በአሁኑ የእርግዝና ወቅት ጫት ቅመሽ ታውቂያለሽ?	1. አዎ<br>2. የለም	
507.	ካሁን በፊት እንደ ሃሺሽ / ሺሻ የመሳሰሉ አደንዛዥ እፆችን/ መድኃኒቶችን ተጠቅመሽ ታውቂያለሽ?	1. በጭራሽ ⟹<br>2. በየቀኑ እጠጣለሁ<br>3. በሳምንት አንዴ ወይም ሁለት ጊዜ<br>4. ሌሎች (ይግለጹ) _______<br>5. መልስ የለም	**601**
508.	ካሁን በፊት እንደ ሃሺሽ / ሺሻ የመሳሰሉ አደንዛዥ እፆችን/ መድኃኒቶችን ተጠቅመሽ የምታውቂ ከሆነ፣ በአሁኑ የእርግዝና ወቅት ሃሺሽ / ሺሻ የመሳሰሉ አደንዛዥ መድኃኒቶችንተጠቅመሽ ታውቂያለሽ?	1. አዎ<br>2. የለም	

Questions from on maternal outcomes during labor and delivery (601 to 804) collected using the English version of the questionnaire (Available in S2_File)

**Data** collection at six weeks postpartum period<br>**ከወሊድ በኋላ ስለሚያጋጥሙ የአዕምሮ ጤና የሚመለከት** የEPDS መጠይቅ<br>በቅርቡ ልጅ ስለወለድሽ፣ ዛሬ ቀን እና ከዛሬ ቀን በፊት በነበሩት 7 ቀናት ውስጥ ምን እንደተሰማሽ ማወቅ እንፈልጋለን		
**ተ.ቁ**	**ጥያቄዎች**	**መልስ**
901.	ባለፉት ሰባት ቀናቶች ውስጥ መሳቅና የነገሮችን አስደሳችነት ማየት ችለዋል?	0. ሁሌ የምችለውን ያህል<br>1. አሁን በጣም ብዙም አይደለም<br>2. በእርግጥ አሁን ብዙም አይደለም<br>3. በጭራሽ አይደለም
902.	ባለፉት ሰባት ቀናቶች ውስጥ ነገሮችን ወደፊት በደስታ ያዩ ነበር?	0. አዎ ሁሌም እንደማደርገው<br>1. በፊት ከማደርገው ያነሰ<br>2. በእርግጥ በፊት ከማደርገው በጣም ያነሰ

		3. በአጠቃሊይ ከባድ ነው
903.	ባለፉት ሰባት ቀናቶች ውስጥ ነገሮች ወደ አላስፈላጊ ሁኔታ ሲያመሩ ያለምክንያት እራስዎን ወቅሰዋል?	3. አዎ ብዙውን ጊዜ<br>2. አዎ አንዳንዴ<br>1. በጣም አንዳንዴ<br>0. አይደለም መቼም ሆኖ አያውቅም
904.	ባለፉት ሰባት ቀናቶች ውስጥ ያለምንም በቂ ምክንያት ተሸብረው ወይም ተጨንቀው ያውቃሉ?	0. አይደለም መቼም ሆኖ አያውቅም<br>1. በጣም አንዳንዴ<br>2. አዎ አንዳንዴ<br>3. አዎ በጣም ብዙ ጊዜ
905.	ባለፉት ሰባት ቀናቶች ውስጥ ያለምንም በቂ ምክንያት የፍርሀትና የድንጋጤ ስሜት ተሰምቶት ያውቃል?	3. አዎ በጣም ብዙ ጊዜ<br>2. አዎ አንዳንዴ<br>1. አይደለም ብዙ ጊዜ አይሰማኝም<br>0. አይደለም በጭራሽ አይሰማኝም
906.	ባለፉት ሰባት ቀናቶች ውስጥ ነገሮች ከቁጥጥርዎ ውጭ ሆነውቦት ያውቃል?	3. አዎ ብዙ ጊዜ ነገሮችን በአጠቃላይ መቋቋም አልችልም<br>2. አዎ ልክ እንደ ሁልጊዜው አንዳንድ ነገሮችን መቋቋም አልችልም<br>1. አይደለም ብዙ ጊዜ በጥሩ ሆኔታ ነገሮችን እቋቋማለሁ<br>0. አይደለም ልክ እንደበፊቱ በጥሩ ሁኔታ ነገሮችን እቋቋማለሁ
907.	ባለፉት ሰባት ቀናቶች ውስጥ በጣም ደስተኛ ባለመሆንዎ እንቅልፍ እምቢ ብልዎት ያውቃል?	3. አዎ በጣም ብዙውን ጊዜ<br>2. አዎ ብዙውን ጊዜ<br>1. በጣም አንዳንድ ጊዜ<br>0. በጭራሽ አይደለም
908.	ባለፉት ሰባት ቀናቶች ውስጥ የሀዘንና የብስጭት ስሜት ተሰምትዎት ያውቃል?	3. አዎ በጣም ብዙውን ጊዜ<br>2. አዎ ብዙውን ጊዜ<br>1. በጣም አንዳንዴ<br>0. በጭራሽ አይደለም
909.	ባለፉት ሰባት ቀናቶች ውስጥ በጣም ከማዘንዎት የተነሳ አልቅሰው ያውቃሉ?	3. አዎ በጣም ብዙ ጊዜ<br>2. አዎ ብዙ ጊዜ<br>1. አልፎ አልፎ ብቻ<br>0. መቼም አይደለም
910.	ባለፉት ሰባት ቀናቶች ውስጥ እራስዎን ለመጉዳት አስበው ያውቃሉ?	3. በጣም ብዙ ጊዜ 2. አንዳንዴ<br>1. በጣም አንዳንዴ 0. በጭራሽ/ መቼም

ስለትብብርዎ በጣም አመሰግናለሁ!!

(**<u>ለመረጃ ሰብሳቢ፡</u>** ከወሊድ በኋላ ስለሚያጋጥሙ የአዕምሮ ጤና የሚመለከት የEPDS መጠይቅ (ክፍል 10) ከተሞላ በኋላ፣ ለጥያቄ 1001-1010 የተሰጡትን መልሶች ቁጥራቸውን ይድምሩ: **ድምር፡ ____/ 30**
የእናቲቱ ውጤት 10 ወይንም ከ10 በላይ ከሆነ፣ እናቲቱ ለተጨማሪ ምርመራ ወደ ቅርብ የጤና ተቋም እንዲሄዱ የምክር አገልግሎት ይስጡ።)
